# On the Treatment of Pneumonia by Restoratives

**Published:** 1865-07

**Authors:** John Hughes Bennett


### SELECTED.

### ON THE TREATMENT OF PNEUMONIA BY RESTORATIVES.

By JOHN HUGHES BENNETT, M. D.

Observing that an effort is being made to restore the dangerous practice of bleeding in pneumonia, it will not be uninteresting to review the results of my experience regarding that disease in the Royal Infirmary of Edinburgh; the more so as the treatment I have pursued is not only most satisfactory, but the result is demonstrated by a series of recorded facts, the accuracy of which will, I think, not be disputed.

Between the 1st of October, 1848, and the 31st of January, 1865, I have been on active duty in the Royal Infirmary seventy-five months, or a computed period of six years and a quarter. During this period I have treated 129 cases of acute pneumonia. Of these, 105 were uncomplicated, and all recovered, although many of them were very severe, involving the whole of one lung in 15, and portions of both in 26 cases. Amongst the 24 complicated cases were 4 deaths: 2 from supervening meningitis, 1 from chronic Bright's disease, and 1 from extensive ulceration of the intestines. Every case has been treated publicly, and is open for inspection in the ward books; and the tabular view of the whole—commenced by Dr. Glen, my former resident physician—has been completed by the labors of Drs. Smart, Buckworth, and Macdonald, my resident physicians for 1863, 1864 and 1865. To these gentlemen I am much indebted for the pains they have taken in determining the following results, the accuracy of which is guaranteed by each of them having revised the facts in succession.

In the four fatal cases, death was evidently caused by complications independent of the pneumonia. To arrive at true statistics with regard to treatment, therefore, it becomes necessary to eliminate them, and to fix our attention on the one hundred and twenty-five cases which recovered.

*Sex.*—Of the one hundred and twenty-five cases, eighty-five were males and forty were females.

*Age.*—The average age of the males was 31½ years. The average age of the females, 28½ years. The average age of both, 30½ years. Between the ages five and fifteen years was one case—a girl; between ten and twenty years, twenty-nine cases—twelve females; between twenty and thirty years, thirty-five cases—eleven females; between thirty and forty years, twenty-three cases—six females; between fifty and sixty years, eleven cases—one female; between sixty and seventy years, one case—a female; and between seventy and eighty years, one case—a female.

*Simple or uncomplicated Pneumonia.*—Of the one hundred and twenty-five cases, there were one hundred and five simple or uncomplicated, and twenty complicated. Of the former, there were seventy-four males and thirty-one females. Seventy-nine were single and twenty-six double cases. Of these I find that the clerk has omitted to state either the exact day of rigor or of convalescence in six, so that no conclusion can be derived from them as to the duration of the disease. Of the remaining ninety-nine cases, seventy-three were single and twenty-six double. The duration of the disease in the seventy-three cases of single uncomplicated pneumonia, counting from the occurrence of rigor to the commencement of convalescence, was as follows: two cases recovered in five days, four cases in seven days, five cases in eight days, two cases in nine days, eight cases in ten days, seven cases in eleven days, seven cases in twelve days, four cases in thirteen days, thirteen cases in fourteen days, two cases in fifteen days, three cases in sixteen days, three cases in seventeen days, three cases in eighteen days, one case in nineteen days, two cases in twenty days, three cases in twenty-one days, one case in twenty two days, two cases in twenty-three days, and one case in twenty-six days; the average duration, 13 2-7 days. The duration of the disease in the twenty-six cases of double uncomplicated pneumonia, counting from the occurrence of the rigor to the commencement of convalescence, was as follows: two cases recovered in eight days, one case in nine days, two cases in ten days, two cases in eleven days, one case in twelve days, one case in thirteen days, four cases in fourteen days, one case in fifteen days, two cases in sixteen days, two cases in eighteen days, two cases in nineteen days, one case in twenty days, three cases in twenty-one days, one case in twenty-seven days, and one case in fifty-five days; the average duration, $16\frac{3}{4}$ days.

Of the one hundred and five simple or uncomplicated cases, there were nine bled by venesection and subjected to an antiphlogistic treatment before or immediately upon admission, before I saw them. The amount of blood extracted varied from twelve to thirty-six ounces; the latter in two bleedings. The duration of one case is not stated. Of the remaining eight cases, the duration was as follows: one case recovered in seven days, two cases in fourteen days, one case in sixteen days, one case in seventeen days, one case in twenty days, one case in twenty-seven days, and one case in fifty five days; the average duration was $21\frac{1}{4}$ days.

The average duration of residence in hospital of the single uncomplicated cases of pneumonia—excluding two cases in which the date of dismission is omitted, making seventy-seven cases—was 21 2-7 days: for the males (fifty-two), 18 3-5 days; and for the females (twenty-five), 27 1-5 days. Of the twenty-six double uncomplicated cases, the average duration of residence in hospital was 23 3-5 days: of the males (twenty), 23 17-20 days; of the females (six), $22\frac{2}{3}$ days. The average duration of residence in hospital of eight cases bled by venesection early in the disease (the ninth case being excluded in consequence of the day of dismission not being entered in the case-book) was thirty-two days.

*Complicated Cases of Pneumonia.*—Of the twenty complicated cases of pneumonia, sixteen were single and four double. Of the sixteen single complicated cases the duration of the disease cannot be determined in three. Of the remaining thirteen, the duration was as follows: One case recovered in seven days, two cases in nine days, one case in ten days, one case in twelve days, two cases in fourteen days, one case in fifteen days, two cases in sixteen days, two cases in nineteen days, and one case in forty-eight days; the average duration, sixteen days. Of the four double cases of complicated pneumonia, one case recovered in nine days, one case in fourteen days, one case in fourteen days, one case in fifteen days, and one case in eighteen days; the average duration, fourteen days.

The extent of pulmonary tissue involved in the pneumonia was carefully determined in each case, and the average duration of the disease in the ninety-five single cases, deducting the unsatisfactory ones, counting from the rigor to the commencement of convalescence, was as follows: One-quarter of the lung (two cases), average duration 8½ days; one-third of the lung (twelve cases), 12 days; one-half of the lung (twenty-five cases), 15¾ days; two-thirds of the lung (thirty-four cases), 14 days; three-fourths of the lung (six cases), 14¾ days; four-fifths of the lung (one case), 12 days; the whole lung (fifteen cases,) 10¾ days. Of these ninety-five cases, the right lung was affected in fifty-eight, the left lung in thirty-seven. Among these ninety-five cases also, the pneumonia was confined to the upper lobe in eleven cases, or nearly one in nine of the whole; and the average duration of the disease in these was thirteen days, and of their residence in the hospital fourteen and a half days.

A careful study of the preceding facts will, I think, tend to establish some new truths and correct several prevailing errors with regard to pneumonia. I must remind those, however, who may yet be sceptical as to the value of a restorative treatment, and imagine that some of these cases might not have been pneumonia at all, that they were all diagnosed and treated publicly in the Royal Infirmary; were all examined, not only by myself, but by intelligent clerks and assistants; and were all made the subject of clinical lectures and commentaries at the bedside. There is, therefore, the positive certainty, not only that every one of these cases was a genuine case of pneumonia, but that no other cases of the disease but what are tabulated were treated by me during the period referred to. It should be explained, however, that a few cases were partly treated by my colleagues, either before I assumed duty or after I left it, in the too frequent rotations which occur among the clinical professors in this University. Such cases are not inserted. It is also necessary to point out that two or three cases brought into the house by the police in an exhausted condition died before I saw them, and are also not inserted. It is the more important to refer to such occurrences, because they serve to account for the differences which must always exist between general hospital and clinical statistics. Every hospital physician must be aware that the general records of the house afford no index whatever to the number of cases of acute pneumonia treated clinically, comprehending as they do not only consecutive, latent, and chronic pneumonias which have entered in a dying condition and have not been treated at all.

1. The first great fact which the preceding figures serve to establish is, that a simple primary pneumonia, whether single or double, if treated by the restorative plan is not a fatal disease. Surely one hundred and five cases, of which twenty-six were double, are sufficient to establish this proposition, especially when it is considered that they were diffused over sixteen years, and occurred in all seasons. Amongst these, also, the whole of one lung was involved in no less than fifteen cases, and the symptoms in many of them were exceedingly severe. Neither will any theory as to strength of constitution or change of type in disease explain the result, as several of the cases were those of healthy, vigorous young laborers, whilst others were those of weak and broken-down seamstresses. In any and every case the disease appears to go through its natural progress so long as the body is not too much exhausted, and the physician as early as possible supports it by nutrients and restoratives.

2. As a general rule, prostration and weakening complications or remedies, not only materially lengthen the period of the disease, but especially prolong the convalescence. It is easily understood, therefore, how it happened that the antiphlogistic treatment of former days proved so fatal. The facts collected for me by Dr. Thorburn, from former case-books of the Royal Infirmary, prove that in weak cases a lowering treatment was still employed, though not perhaps to so great an extent as in robust persons.

3. It is generally supposed that the extent of the disease and the amount of lung affected must influence the result and duration of the disease. As to the result, all my cases recovered, even the fifteen cases where the whole of the lung was involved, as well as the twenty six cases where portions of both lungs were affected. In one complicated case, the whole lung on the right side, and two-thirds of the lung on the left side, were simultaneously affected, thus leaving only one-third of a lung to respire with; and yet, without bleeding, and with the aid of nutrients and restoratives, she was convalescent on the fourteenth day, and left the house quite well after a sixteen days' residence. With regard to duration, the extent of the disease does not exert so much influence as is generally supposed. If only a fourth of one lung be affected, the recovery may take place in eight days; but after that, whether the half or the whole of one lung, or two-thirds of both lungs be affected, it does not appear to cause much difference. Cases with half a lung pneumonic recovered in fifteen, with two-thirds of a lung in fourteen, with a whole lung in ten, and with portions of both lungs in fourteen days on the average.

4. Since the observations of Louis, it has been supposed that a pneumonia at the apex of a lung was more fatal and more prolonged than one at the base; and so it may be with an antiphlogistic treatment. But, with a restorative treatment, the preceding facts show that in eleven cases where the disease was confined to the apex, recovery took place in all, on an average, on the thirteenth day.

5. As means of palliating symptoms, and especially pain and dyspnœa, warm fomentations and poultices I believe to be the best and safest. Chloroform has been given by Varentrapp and others with good effect. No doubt also small bleedings, to the extent of eight or twelve ounces, give relief; but in debilitated persons are dangerous, and in all tend, by weakening the strength at a period when the depressed system is struggling to regain its equilibrium, to prolong the convalescence and favor dangerous sequelæ. Still a small bleeding may be employed as a palliative, with caution, to relieve engorgement of the lungs and congestion of the right side of the heart, although it is very rarely required. It should be remembered, in cases of double pneumonia, that there is often great dyspnœa on the sixth or seventh day, which will generally yield to warm poultices locally, and moderate doses of wine.

6. As a curative treatment, I am satisfied that the best plan is rest in bed, nutritive drinks, especially good beef-tea, from the first, assisted by port wine—from four to eight ounces—if the pulse become weak, and solid nutrients as soon as they can be taken. The elimination of the exudation may be further assisted by salines (acetate of ammonia and small doses of tartar emetic—one sixteenth of a grain) and diuretics (nitric ether), although nature will accomplish this herself if the strength of the body be maintained. All active purgatives, contra-stimulants, depressants, anodynes, and lowering remedies of every description should be avoided.

After carefully studying all that has been written on the subject of pneumonia, as well as the numerous statistical tables of the results of various kinds of practice, I can only account for the remarkable success which has followed the restorative treatment in my hands by supposing that acute pneumonia is not a fatal disease, if the strength be supported and there be no complication. The former idea of medical practitioners, that it was a dangerous disorder, and required active depletion and antiphlogistics to prevent its passing into suppurative or fatal stage, was erroneous, and the result of the imperfect knowledge of pathology which then existed. Suppuration, so far from being fatal, is, as we have endeavored to show, necessary to the resolution of the disease; and a fatal result, so far from being avoided, was produced to the extent of one in every three cases. The late Dr. Todd, while he supported, also stimulated to a great extent, and the result of his practice was a mortality of one in nine cases. In not one of my uncomplicated cases has there, I repeat, been a death for sixteen years, although twenty-six were double cases, and fifteen were cases in which the whole lung was involved. Why, therefore, any such case should have died in the practice of Dr. Todd, I can only ascribe to unnecessary stimulation, as that seems to be only point in which his practice differed from it.

In an able article in the *British and Foreign Medico-Chirurgical Review* for July, 1858, it was endeavored to be shown, from the contrary results obtained by statistics, that the result was governed by hygienic laws or peculiarities such as age, season, climate, etc. I consider that my cases prove the fallacy of such reasoning; and that, looking at the time over which they extend, as well as all the other circumstances which are adverted to, it might easily be shown that the uniform good results in my uncomplicated cases depend on some other cause. That cause, I can have no doubt, is simply supporting the patient by nutrients and restoratives from the commencement. It is the want of that support which explains mortality in the practice of those who, while they do not actually lower their patients, fail to see that, in a certain proportion of cases, either the disease itself or excessive stimulation exhausts and proves fatal.

The facts on which, what appear to me these important conclusions rest, I hope to lay before the profession ere long in a more extended form.

